# Transcriptome and physiological analysis of increase in drought stress tolerance by melatonin in tomato

**DOI:** 10.1371/journal.pone.0267594

**Published:** 2022-05-17

**Authors:** Lu Yang, Sijia Bu, Shengxue Zhao, Ning Wang, Jiaxin Xiao, Feng He, Xuan Gao

**Affiliations:** Key Laboratory for the Conservation and Utilization of Important Biological Resources, College of Life Sciences, Anhui Normal University, Wuhu, Anhui Province, People’s Republic of China; United Arab Emirates University, UNITED ARAB EMIRATES

## Abstract

Drought stress seriously affects tomato growth, yield and quality. Previous reports have pointed out that melatonin (MT) can alleviate drought stress damage to tomato. To better understand the possible physiological and molecular mechanisms, chlorophyll fluorescence parameters and leaf transcriptome profiles were analyzed in the “Micro Tom” tomato cultivar with or without melatonin irrigation under normal and drought conditions. Polyethylene glycol 6000 (PEG6000) simulated continuous drought treatment reduced plant height, but melatonin treatment improved plant growth rate. Physiological parameter measurements revealed that the drought-induced decreases in maximum efficiency of photosystem II (PSII) photochemistry, the effective quantum yield of PSII, electron transfer rate, and photochemical quenching value caused by PEG6000 treatment were alleviated by melatonin treatment, which suggests a protective effect of melatonin on PSII. Comparative transcriptome analysis identified 447, 3982, 4526 and 3258 differentially expressed genes (DEGs) in the comparative groups plus-melatonin *vs*. minus-melatonin (no drought), drought *vs*. no drought (minus-melatonin), drought *vs*. no drought (melatonin) and plus-melatonin *vs*. minus-melatonin (drought), respectively. Gene Ontology and Kyoto Encyclopedia of Genes and Genomes analysis revealed that DEGs in the four comparative groups were involved in multiple metabolic processes and closely related to hormone signal transduction and transcription factors. Transcriptome data revealed that melatonin changed the expression pattern of most hormone signal transduction related DEGs induced by drought, and improved plant drought resistance by down-regulating the expression of linoleic acid catabolic enzyme genes. These results provide new insights into a probable mechanism of the melatonin-induced protection of photosynthesis and enhancement of drought tolerance in tomato plants.

## Introduction

With the deterioration of climatic and environmental conditions, global crop plant production is suffering from severe abiotic stresses, such as cold, drought and salt stress [1]. Drought is one of the most commonly encountered stress environments affecting crop yield and quality. High-temperature stress caused by global warming intensifies evaporation from soil horizontal, whereas altered precipitation patterns result in drought stress as a result of insufficient rainfall, causing reduced crop yield [2–4]. The loss of agricultural production caused by drought is greatest in China [5].

Tomato (*Solanum lycopersicum* L.) is an economically important crop, cultivated widely throughout the world. It is rich in nutrients and is an important model plant for studying the ripening of fleshy fruits [6]. Drought stress seriously affects the growth and development of tomato, resulting in large-scale yield reductions [7, 8]. Previous studies have shown that drought stress can change the physiological and biochemical characteristics of tomato leaves, causes damage to the photosynthetic system, reduces plant growth rate and dry matter accumulation, and affects flowering, fruit setting and ripening [9–11].

Polyethylene glycol 6000 (PEG6000) is a hydrophilic macromolecule with a molecular mass of 6000 Da. When PEG6000 is applied in the irrigation water, it is difficult for plant roots to absorb water from the soil because the water potential of the PEG6000 solution is low. Therefore, PEG6000 is often used as an agent to simulate drought experimentally [12]. Melatonin (MT), also known as N-acetyl-5-methoxytryptamine, belongs to the indole tryptamines. It can promote vegetative growth of horticultural plants, inhibit reproductive growth, regulate photoperiod, regulate root development, delay leaf senescence, and affect fruit ripening and storage [13]. Also, many studies have shown that melatonin is an active antioxidant, involved in the regulation of abiotic and biotic stress tolerance of horticultural crops. For example, under drought stress, exogenous melatonin can activate the antioxidant system of apple, removing excess reactive oxygen species (ROS), improving the efficiency of light energy conversion and carbon assimilation in leaves, and then alleviating drought symptoms [14]. The photosynthetic rate and drought tolerance of cucumber seedlings grown under osmotic stress were significantly increased by exogenous melatonin treatment [15]. Melatonin treatment can improve thermotolerance in several plant species such as rice, *Arabidopsis*, tomato, and tall fescue [16–19]. In maize, exogenous melatonin application enhanced salt stress tolerance by improving antioxidant and photosynthetic capacity, and melatonin can also exert a protective role against drought stress [20, 21]. Additionally, damage caused by cold stress can be alleviated by melatonin treatment of *Arabidopsis thaliana* and cucumber [22, 23]. For abiotic stress, melatonin can induce disease resistance to the fungal pathogen *Botrytis cinerea* in tomato fruit by activating the jasmonic acid signaling pathway [24]. Exogenous application of melatonin can also contribute to pathogen resistance in apple (to the fungal pathogen *Diplocarpon mali*), Lupinus albus (*Penicillium spp*.) and *Arabidopsis* (to the bacterial pathogen *Pseudomonas syringae* pv. tomato (Pst) DC3000) [25–27].

In tomato, melatonin application can affect plant growth and fruit ripening [28, 29]. Melatonin treatment also mitigated chilling-induced oxidative stress and photosynthetic inhibition, and alleviated heat-induced damage to tomato seedlings by balancing redox homeostasis and modulating polyamine and nitric oxide biosynthesis [30]. Overexpression of the melatonin synthesis-related tomato gene *SlCOMT1* improved the tolerance of tomato to salt stress [31]. With respect to drought-stressed plants, studies showed that melatonin treatment enhanced the photosynthetic performance and antioxidant capacity of tomato seedlings [32]. Melatonin can improve plant stress tolerance by inhibiting ROS production in chloroplasts and by regulating photosynthetic electron transport and D1 protein synthesis [32]. From the cytological point of view, previous studies had shown that, in tomato plants, exogenous melatonin application improved tolerance to water deficit by promoting cuticle formation [33]. A recent study showed that exogenous melatonin application could help alleviate deleterious effects of PEG (simulating drought) by enhancing antioxidant mediated defense and by increasing the concentrations of some phenolics [34]. Recently, a study highlighted the positive effect of melatonin in open-field droughted tomato plants by enhancing the quantity and quality of strategic crops [35].

Despite these multiple physiological and biochemical effects of melatonin, changes in gene expression levels in tomato in response to melatonin irrigation in the presence of PEG6000 have not yet been elucidated. In the present study, RNA-sequencing (RNA-Seq) technology was used to examine the effect of the response to melatonin irrigation in normal and drought-stressed plants. Combined with the analysis of chloroplast fluorescence parameters, such as maximum efficiency of photosystem II (PSII) photochemistry (Fv/Fm), the effective quantum yield of PSII (Fv’/Fm’), electron transfer rate (ETR), photochemical quenching (qP), non-photochemical quenching coefficient (qN) and stern-volmer nonphotochemical quenching (NPQ), the analysis of plant phenotype and differentially expressed genes (DEGs) will lay the foundation for further explanation of the role of melatonin in inducing drought tolerance in tomato.

## Materials and methods

### Plant materials and treatments

*Solanum lycopersicum* L. cv. Micro-Tom was selected as the wild type (WT). Tomato plants were grown in a greenhouse with standard conditions (18 h light/6 h dark, 25°C day/18°C night, and 60% relative humidity) and watered daily. In our experiment, one month old tomato plants with the same growth potential were selected which were cultivated on imported peat with weak acidity and pH 5.5. The experiment was divided into control group (without PEG6000 treatment) and drought experimental group (100 mM PEG6000 treatment). Three melatonin treatment concentrations (0 μmoL/L、10 μmoL/L、50 μmoL/L) were set in each group. Specifically, in control group (CM), CM0 represented PEG6000 (−) and melatonin (−) treatment, CM10 represented PEG6000 (−) and melatonin (10 μM) treatment, and CM50 represented PEG6000 (−) and melatonin (50 μM) treatment. In experimental group (TM), TM0 represented PEG6000 (100 mM) and melatonin (−) treatment, TM10 represented PEG6000 (100 mM) and melatonin (10 μM) treatment, and TM50 represented PEG6000 (100 mM) and melatonin (50 μM) treatment. Then, root irrigation was used and the treatment time and position were fixed. In detail, the prepared solution of PEG6000 or melatonin was uniformly and slowly watered from the right side of the plant without touching the stems and leaves and the treatment time was fixed at 5:00 p.m. Three tomato plants were selected as biological repeats for each concentration and following treatment was then carried out: (1) The tomato seedlings in the experimental group were treated with 50 mL PEG6000 solution of 100 mmol/L, and the control group were treated with 50 mL ddH_2_O; (2) The next day, the tomato seedlings of the experimental group and the control group were treated with 50 mL melatonin solution of gradient concentration (0 μmol/L, 10 μmol/L, 50 μmol/L) respectively. (3) PEG6000 and melatonin solution were performed alternately and two days treatment was a cycle, the treatment was finished after eight cycles (16 days).

### Chlorophyll fluorescence parameters

A portable fluorometer was used at room temperature to measure chlorophyll fluorescence parameters of fully expanded tomato leaves, at least three plants were used in each treatment. After full dark adapted within two hours, all the electronic gates were in the open state, the minimum fluorescence yield at the dark-adapted state (F0) was recorded. Then a saturation pulse of 3000 μmol (photon) m^-2^ s^-1^ for 3 s was given, at this time, all the electronic gates convert the energy used for photosynthesis into fluorescence and heat, and the maximal fluorescence yield at the dark-adapted state (Fm) was recorded. After that, the maximal (Fm′) and minimum (F0′) fluorescence yield at the light adapted state, steady state (Fs) fluorescence yield at the light adapted state were measured with tomato leaves treated with an active light of 1000 μmol (photon)·m^-2^ s^-1^. Fv represented variable fluorescence at the dark-adapted state. Maximum efficiency of PSII photochemistry was calculated as Fv/Fm, where Fv = Fm–F0; the effective quantum yield of PSII was calculated as Fv’/Fm’, where Fv’ = Fm’ − F0’; and qP = (Fm’–Fs)/(Fm’ − F0’) were determined in the light adapted state. And also, Stern-Volmer nonphotochemical quenching (NPQ, NPQ = (Fm − Fm’)/Fm’) together with electron transfer rate (ETR, ETR = Φ PS Ⅱ × i × a × f, in which i, a and f represents intensity of incident light, absorption ratio of incident light, and estimated value of F-energy distribution proportion, respectively) were also calculated and recorded [36–38].

### Preparation of RNA-Seq libraries and sequencing of tomato

After treatment, leaves of tomato plant with and without melatonin irrigation under normal and PEG6000 treatment conditions were collected. For each group, two biological replicates were carried out, total RNA was extracted using Trizol reagent (Invitrogen) and then treated with RNase-free DNase I (Thermo, USA) to remove contaminating genomic DNA according to the manufacturer’s instructions. cDNA libraries were then constructed for sequencing on the DNBSEQ platform (BGI, China). Clean data were obtained by removing reads containing adapters and low-quality reads from the raw data. All subsequent analyses were based on clean, high-quality data. The average blast ratio of sample to genome was 97.52% and the average matching rate was 86.56%. A total of 27738 genes were detected successfully. All sequencing reads were uploaded to the National Center for Biotechnology Information (SRA accession number: PRJNA812356).

### Transcriptome analyses of tomato leaves

Given that the tomato genome data has been published, the reference genome version is IATG 4.0_release. After grouping multiple samples of biological duplication into a group, the types of pairwise comparisons were as follows: 10 μM melatonin irrigation group compared to 0 μM melatonin irrigation group, both groups performed without PEG6000 treatment (CM10-vs-CM0); 10 μM melatonin irrigation group compared to 0 μM melatonin irrigation group, both groups performed with 100 mM PEG6000 treatment (TM10-vs-TM0); 100 mM PEG6000 treatment group compared to 0 mM PEG6000 treatment group, both groups performed without melatonin irrigation (TM0-vs-CM0); 100 mM PEG6000 treatment group compared to 0 mM PEG6000 treatment group, both groups performed with 10 μM melatonin irrigation (TM10-vs-CM10). For difference analysis of gene expression between two groups, the method of DEG-seq was used based on Poisson distribution [39]. In order to improve the accuracy of differentially expressed genes (DEGs), genes with more than two times of difference and Q-value ≤ 0.001 were defined as the significantly differentially expressed genes. For DEGs in multiple comparison groups, Venn diagrams was used for displaying. For GO enrichment, the DEGs were classified into several functional groups according to GO annotation results and official classification method. At the same time, the Phyper function in R software was used for enrichment analysis, then p value was calculated and FDR correction was performed. When the corrected p value (Qvalue) ≤ 0.05, it was considered that the GO function was significantly enriched. According to the KEGG pathway annotation classification, the Phyper function in R software was used for enrichment analysis, p value was calculated and then corrected by FDR. Similarly, the function with Qvalue ≤ 0.05 is regarded as significant enrichment. Flow chart of transcriptome analysis could be found in S1 Fig.

### Verification of DEGs identified from RNA-Seq by qPCR

Fourteen randomly selected DEGs originated from RNA-Seq data were verified by qRT-PCR. Firstly, total RNAs were extracted using Plant RNA Extraction Kit following the protocol provided by the manufacturer (Tiangen, China). About 2 μg of total RNA from each sample was used for first-strand cDNA synthesis (NovoScript, China). For quantitative real-time PCR (qPCR), *Slactin* was used as internal control and the reaction was performed with SuperReal PreMix Plus (SYBR Green) (Tiangen, China). Gene-specific primers were designed with the software of DNAMAN8 and 20 μL reaction system contained 1.0 μL of cDNA, 1.2 μL of primers, 10 μL of 2×SuperReal PreMix Plus, and 7.8 μL of distilled water. The procedures of PCR amplification consisted of an initial incubation at 95°C for 15 min, followed by 40 cycles of 95°C for 10 s and 60°C for 30 s with Bio-Rad CFX connect (Bio-Rad) and melting curve analysis was performed ranging 60–95°C. Each sample was amplified in triplicate and the cycle threshold (Ct) 2^-Δ(ΔCt)^ method was adopted for relative quantification of specific mRNA levels. All primers for qPCR are listed in S1 Table.

## Results

### Plant growth

**Fig 1:**
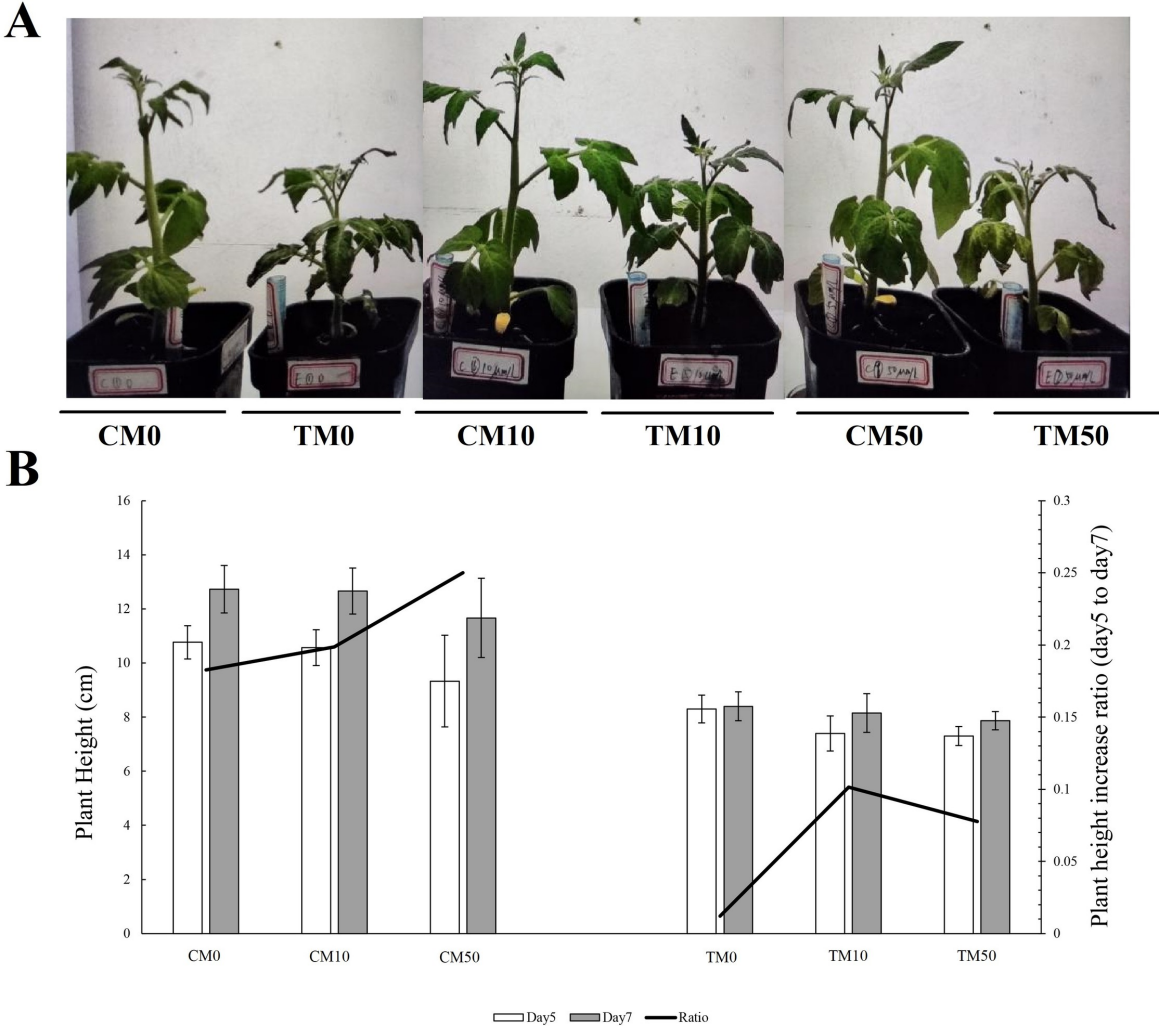
Melatonin alleviates tomato plant damage induced by PEG6000 simulated drought treatment. A. Growth state of tomato plants after combined application of different concentrations of melatonin (0, 10, 50 μmol/L) under normal and PEG6000 simulated drought conditions. CM0, melatonin was not applied under normal condition; TM0, melatonin was not applied under PEG6000 simulated drought condition; CM10, 10 μmol/L melatonin was applied under normal condition; TM10, 10 μmol/L melatonin was applied under PEG6000 simulated drought condition; CM50, 50 μmol/L melatonin was applied under normal condition; TM50, 50 μmol/L melatonin was applied under PEG6000 simulated drought condition. B. Plant height and plant height increase ratio (day 5 to day 7) were measured on the fifth and seventh days after treatment. Data represent mean values ± SD and are derived from at least three plants.

Here, the PEG6000-treated group and the non-PEG6000-treated group were described as the experimental group (TM) and the control group (CM), respectively. As shown in Fig 1A, the height of plants in the experimental group was shorter than that of the control group, regardless of whether melatonin (0, 10, 50 μmol/L) was applied or not. Interestingly, in the experimental group, compared with the experimental plants grown without melatonin (TM0), 10 μmol/L melatonin (TM10) alleviated leaf wilting. However, the effect of 50 μmol/L melatonin (TM50) was not obvious (Fig 1A). The height of plants in the experimental groups (TM0, TM10, TM50) were clearly lower than that of the control group (CM0, CM10, CM50), whether measured on the fifth or seventh days. During the processing cycle from day 5 to day 7, in the control group, the percentage increase in height of CM0, CM10 and CM50 plants was 18.27%, 19.87% and 25.00%, respectively; and in experimental group, the corresponding increases of TM0, TM10 and TM50 were 1.20%, 10.13% and 7.76%, respectively. In other word, in either the control or experimental groups, plant height increased ratio was greater after applying melatonin solution. Interestingly, in the experimental group, the percentage plant height increase was greatest with the application of 10 μmol/L melatonin, compared with 0 or 50 μmol/L melatonin (Fig 1B).

### Photosynthetic performance

**Fig 2:**
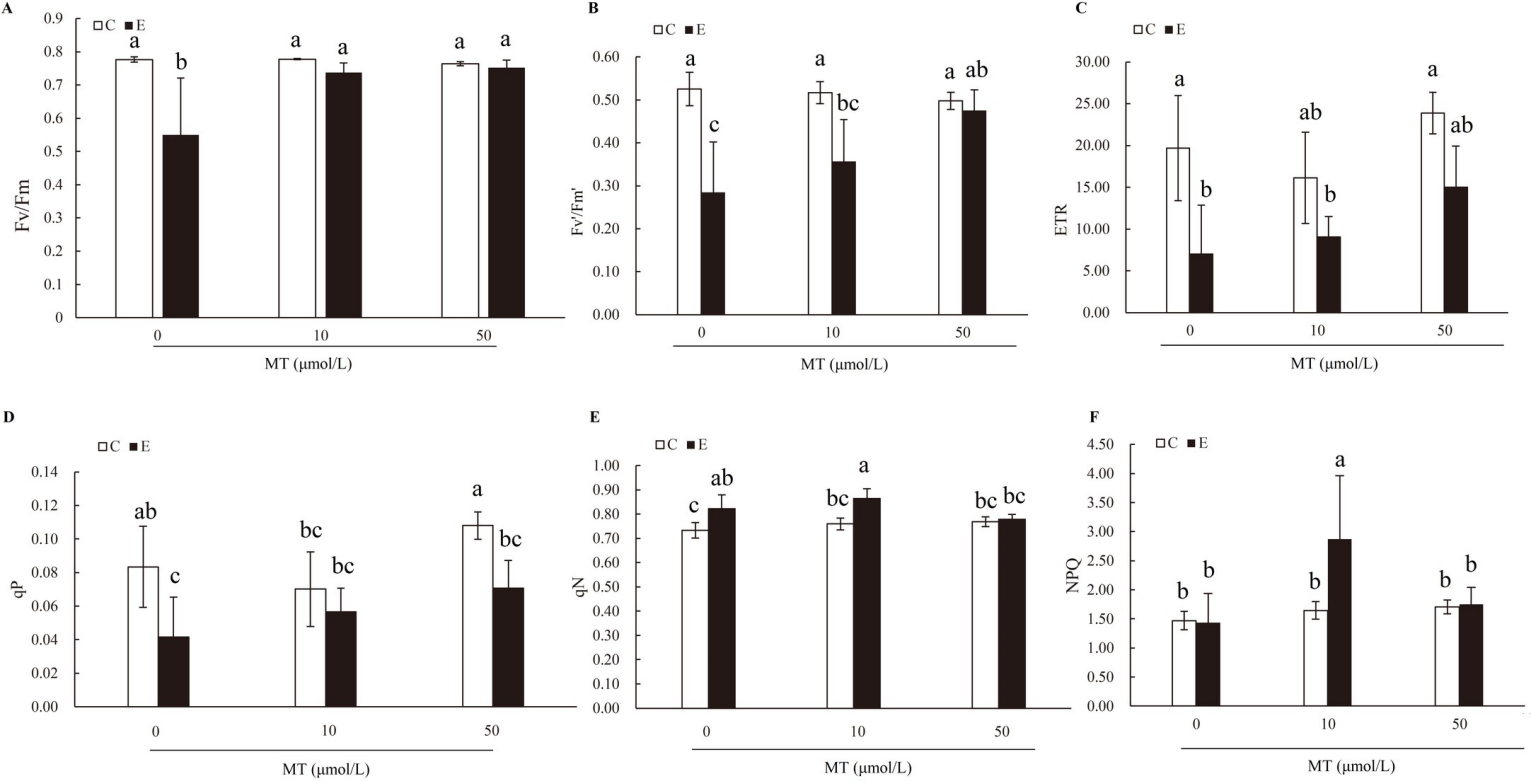
Melatonin alleviated the photosynthetic damage of tomato leaves under PEG6000 simulated drought treatment. Maximum photochemistry efficiency of photosystem (PS)II (Fv/Fm) (A), effective quantum yield of PSII photochemistry (Fv′/Fm′) (B), electron transfer rate (ETR) (C), photochemical quenching (qP) (D), non-photochemical quenching coefficient (qN) (E), and non-photochemical quenching (NPQ) (F) of tomato leaves after combined application of different concentrations of melatonin (0, 10, 50 μmol/L) under normal and PEG6000 simulated drought. The experiments were repeated three times for each and the statistical data are presented as the mean ± standard deviation. Differences were compared by Duncan’s test with a significance level of p < 0.05.

The Fv/Fm, Fv′/Fm′, ETR, qP, qN and NPQ were measured and compared between the experimental and control groups. Without melatonin treatment, the Fv/Fm, Fv′/Fm′, ETR, and qP of tomato plants decreased in the stressed plants of the experimental group when compared with the non-stressed plants of the control group, which indicated that PSII was damaged. After melatonin irrigation, irrespective of the concentration (10 or 50 μmol/L), the decrease in Fv/Fm, Fv′/Fm′, ETR, and qP value caused by stimulated drought stress in the experimental group was greatly diminished (Fig 2A–2D). The effect of melatonin on qN was not significant, which suggested that melatonin had no obvious effect on the heat loss of plants to tolerate drought stress (Fig 2E). Melatonin (10 μmol/L) increased NPQ, whereas the effect was not significant with 50 μmol/L melatonin treatment (Fig 2F).

### Identification of differentially expressed genes

**Fig 3:**
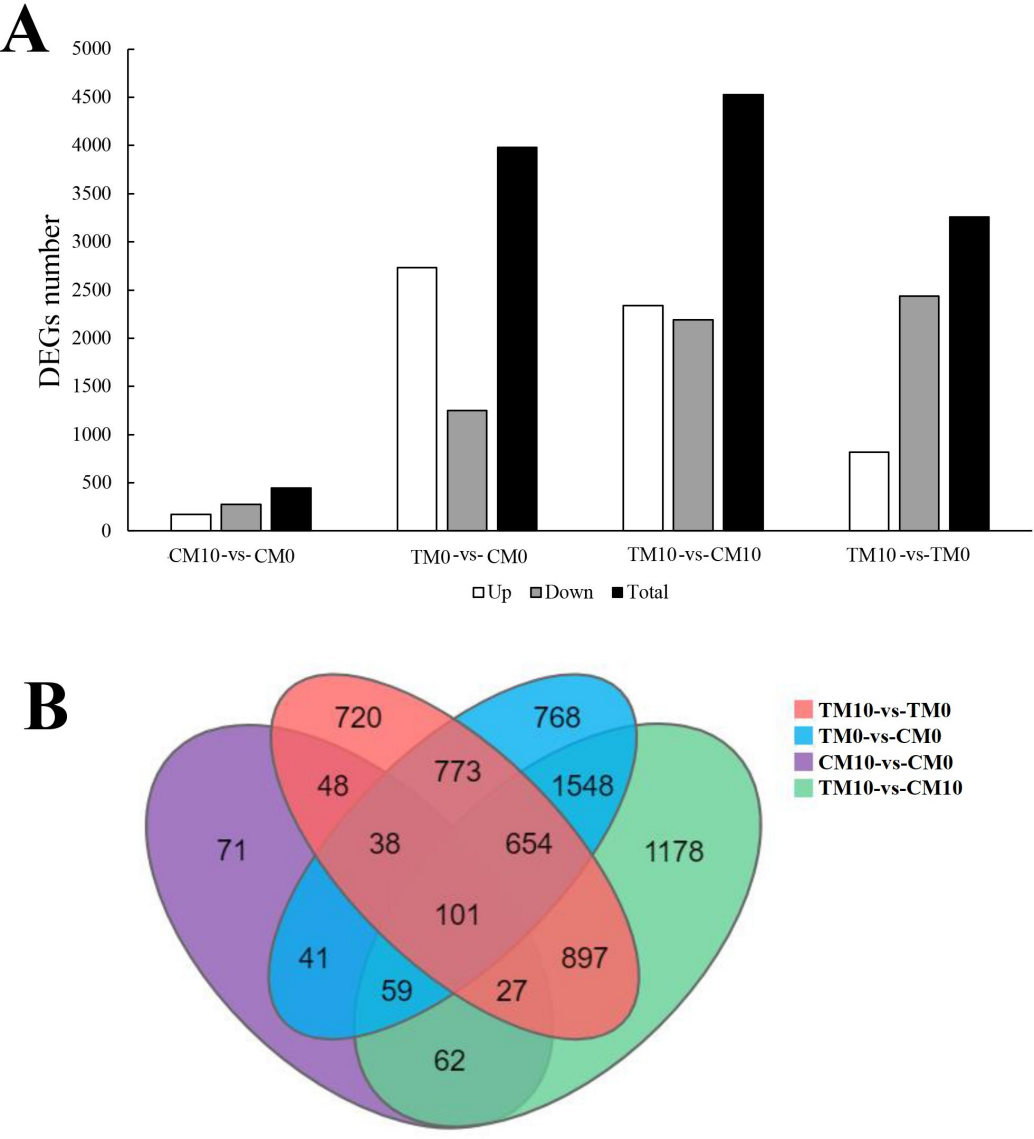
The number of DEGs in the four comparison groups (CM10-vs-CM0, TM0-vs-CM0, TM10-vs-CM10, TM10-vs-TM0) and venn diagram analysis. A. The total DEGs number and DEGs numbers of up-regulated as well as down-regulated genes in the four groups. B. Venn diagram analysis of the DEGs numbers in the four groups.

Transcriptome analysis of tomato leaves treated with 0 μmol/L (CM0) or 10 μmol/L (CM10) melatonin in the control (non-stressed) group and 0 μmol/L (TM0) or 10 μmol/L (TM10) melatonin in the experimental (drought-stressed) group was performed. Without exposure to drought, a total of 447 DEGs were found in tomato leaves treated with 10 μmol/L (CM10) melatonin, when compared with those treated with 0 μmol/L (CM0) melatonin, including 174 up-regulated genes and 273 down-regulated genes (CM10-vs-CM0) (Fig 3A, S2 Table). In the presence of drought stress, however, a total of 3258 DEGs were found in tomato leaves treated with 10 μmol/L(TM10) melatonin, when compared with those treated with 0 μmol/L (TM0) melatonin, namely 819 up-regulated genes and 2439 down-regulated genes (TM10-vs-TM0) (Fig 3A, S3 Table). In the absence of melatonin, a total of 3982 DEGs were identified in drought-stressed plants, namely 2734 up-regulated genes and 1248 down-regulated genes (TM0-vs-CM0) (Fig 3A, S4 Table); after treatment with 10 μmol/L melatonin, a total of 4526 DEGs were identified in drought-stressed plants, namely 2337 up-regulated genes and 2189 down-regulated genes (TM10-vs-CM10) (Fig 3A, S5 Table). Therefore, the largest number of DEGs existed between TM10 and CM10 (TM10-vs-CM10), whereas the smallest number occurred between CM10 and CM0 (CM10-vs-CM0). The distribution of up-regulated and down-regulated genes is shown using a volcano plot (S2 Fig). Furthermore, DEGs among the four comparison groups were analyzed by venn diagram and results showed that a total of 101 DEGs existed in common (Fig 3B).

### GO and KEGG analyses of DEGs in the four comparison groups

The DEGs in the four comparison groups (CM10-vs-CM0, TM10-vs-TM0, TM0-vs-CM0, TM10-vs-CM10) were conducted Gene Ontology (GO) and Kyoto Encyclopedia of Genes and Genomes (KEGG) enrichment analysis.

**Fig 4:**
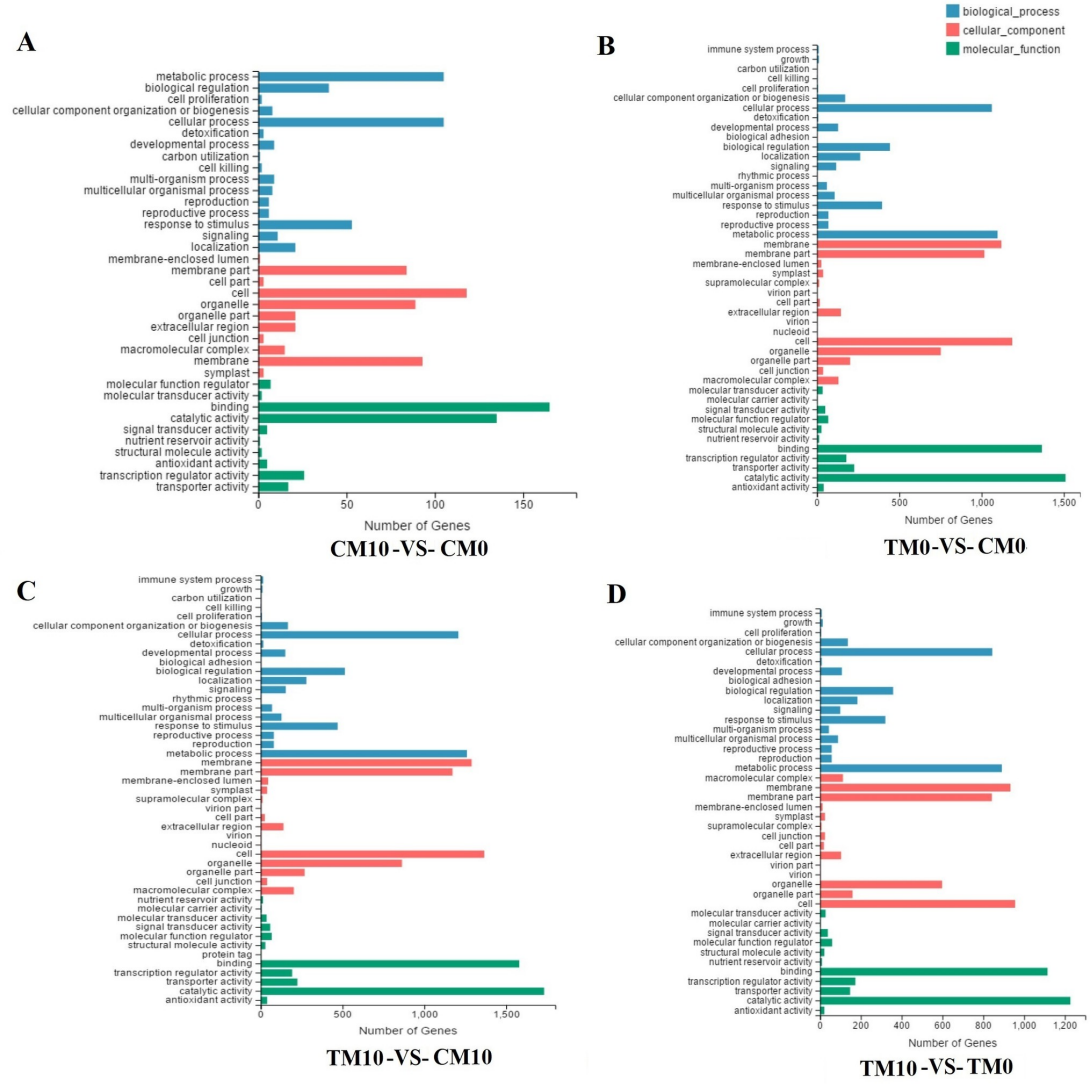
Function annotation of DEGs in the four comparison groups (CM10-vs-CM0, TM0-vs-CM0, TM10-vs-CM10, TM10-vs-TM0) by Gene Ontology (GO) analysis. A. GO analysis of DEGs in the comparison group of CM10-vs-CM0. B. GO analysis of DEGs in the comparison group of TM0-vs-CM0. C. GO analysis of DEGs in the comparison group of TM10-vs-CM10. D. GO analysis of DEGs in the comparison group of TM10-vs-TM0.

**Fig 5:**
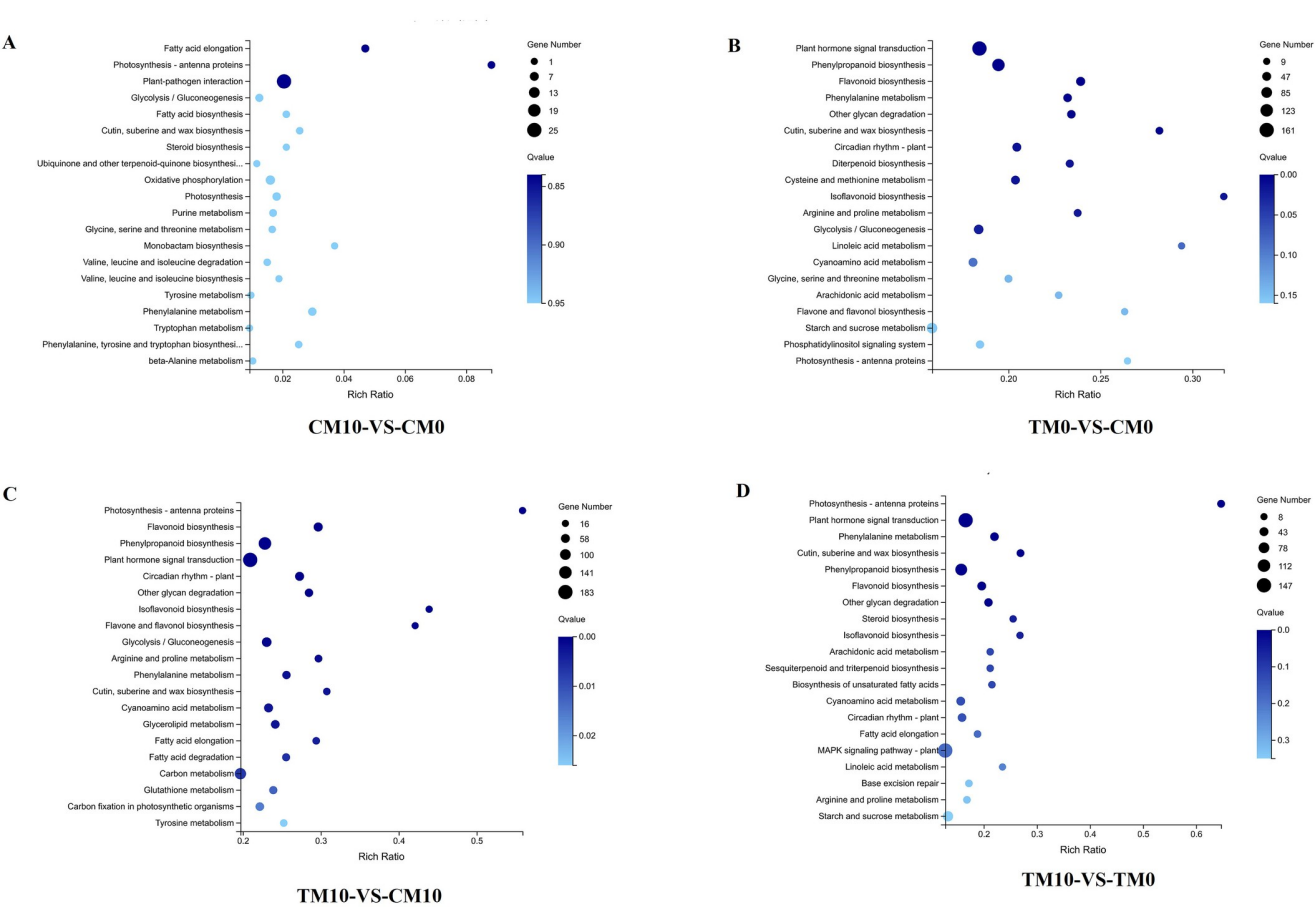
Enrichment bubble diagram in KEGG pathway of DEGs in the four groups (CM10-vs-CM0, TM0-vs-CM0, TM10-vs-CM10, TM10-vs-TM0). The enrichment of KEGG pathway showed with bubble diagrams from three dimensions. The default is the first 20 GO terms with the lowest Qvalue or the selected GO terms (sorted by Qvalue, up to 60). A. KEGG pathway enrichment bubble diagram in the comparison group of CM10-vs-CM0. B. KEGG pathway enrichment bubble diagram in the comparison group of TM0-vs-CM0. C. KEGG pathway enrichment bubble diagram in the comparison group of TM10-vs-CM10. D. KEGG pathway enrichment bubble diagram in the comparison group of TM10-vs-TM0.

For GO analysis, the DEGs in the CM10-vs-CM0 group were classified into 37 functional terms, namely 16 terms in BP, 11 terms in CC, and ten terms in MF. Within BP, cellular processes and metabolic processes were predominant. Within the CC domain, cell, membrane, membrane part and organelle represented the majority of DEGs in this category. For MF, binding and catalytic activity were the most abundantly assigned terms (Fig 4A). The total DEGs between TM0-vs-CM0 were classified into 46 functional terms, namely 20 terms in BP, 15 terms in CC, and 11 terms in MF. Within BP, cellular process and metabolic process were the dominant subcategories. Within the CC domain, cell, membrane, membrane part and organelle represented the majority of terms in this category. For MF, binding and catalytic activity were the most abundantly assigned terms (Fig 4B). The DEGs of TM10-vs-CM10 were classified into 47 functional terms, namely 20 terms in BP, 15 terms in CC, and 12 terms in MF. Within the BP domain, cellular process and metabolic process were the dominant terms. Within the CC domain, cell, membrane, membrane part and organelle represented the majority of the DEGs. For the MF domain, binding and catalytic activity were the most abundantly assigned DEGs (Fig 4C). The DEGs of TM10-vs-TM0 were classified into 42 functional terms, namely 17 terms in BP, 14 terms in CC, and 11 terms in MF. Within the BP domain, cellular process and metabolic process were the dominant subcategories. Within the CC domain, cell, membrane, membrane part and organelle represented the majority of the DEGs. For the MF domain, binding and catalytic activity were the most abundantly assigned DEGs (Fig 4D). For KEGG analysis, the classification and enrichment of DEGs are represented by column diagram (S3 Fig) and bubble diagram (Fig 5), respectively. The highest number of DEGs among the four groups were all involved in metabolism especially in the term of global and overview maps (S3 Fig). Enrichment bubble diagram of DEGs involved in KEGG pathway showed that under drought treatment, the DEGs between melatonin (TM10) and non-melatonin (TM0) treatment revealed much noteworthy information about the effect of melatonin. DEGs with the greatest expression changes in TM10 vs TM0 could be found in S6 Table. As shown in Fig 5D, the DEGs in TM10-vs-TM0 group were mainly enriched in “plant hormone signal transduction” and “photosynthesis-antenna proteins”. Interestingly, term of “linoleic acid metabolism” could be found only in the comparison groups of TM0-vs-CM0 (Fig 5B) and TM10-vs-TM0 (Fig 5D).

### Analysis of DEGs involved in alpha-linolenic acid pathway

**Fig 6:**
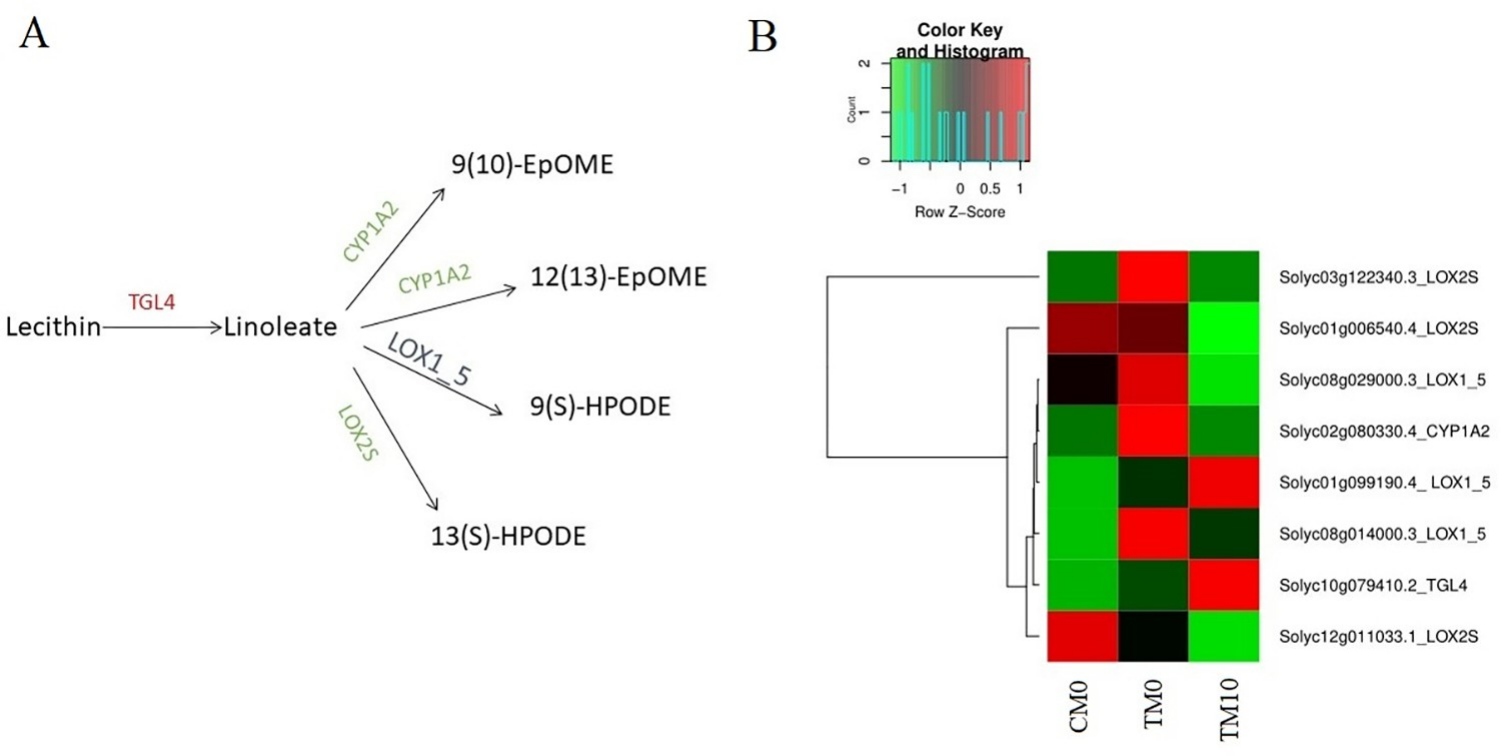
Effects of exogenous melatonin on alpha-linolenic acid metabolism in tomato under drought stress. (A) the alpha-linolenic acid metabolism pathway induced by melatonin in tomato under drought stress (TM10-vs-TM0). The enzymes marked in red and green indicates melatonin-induced up and down regulated genes, respectively. Also, the enzymes marked in dark blue indicates melatonin-induced both up-regulated and down-regulated genes. (B) The expression profiles of the DEGs related to alpha-linolenic acid metabolism in groups of CM0, TM0 and TM10.

According to above enrichment results of DEGs in KEGG pathway, effects of exogenic melatonin on alpha linolenic acid metabolism in tomato under drought stress was focused on (Fig 6). In general, when compared with TM0, DEGs related to linoleic acid synthesis (TGL4) and catabolism related enzymes (CYP1A2 and LOX2S) in TM10 were -up and -down regulated, respectively (Fig 6A). These results indicated that the content of linoleic acid may increase in tomato plant under melatonin treatment to help adaption to drought stress. More specifically, the speculation was confirmed in Fig 6B: when compared with normal condition (CM0), DEGs related to linoleic acid catabolic enzymes were nearly up-regulated under drought treatment (TM0); interestingly, after melatonin irrigation (TM10), the up-regulated expression pattern of DEGs involved in linoleic acid catabolic enzymes was restored to a great extent (Fig 6B).

### Classification of DEGs according to hormones

Classification of DEGs according to hormones in the four comparison groups of CM10-vs-CM0, TM0-vs-CM0, TM10-vs-CM10 and TM10-vs-TM0 were analyzed (S7–S10 Tables). For example, under non-drought conditions, compared with no application of melatonin (CM0), there were 13 ethylene (Eth)-related genes, six auxin (IAA)-related genes, three abscisic acid (ABA)-related genes, two cytokinin (CK)-related genes, one gibberellin (GA)-related gene and two salicylic acid (SA)-related genes out of 447 DEGs in the group of CM10, of which *Solyc05g024260*.*3* was involved in both the ABA and SA metabolic pathways (S7 Table). Among the 101 DEGs common to the above four groups, ten were related to hormones and five were related to ethylene; more detailed information can be found in Table 1.

**Table 1:** Hormone related DEGs shared by four groups.

Gene ID	hormone	log2(CM10/CM0)	log2(TM0/CM0)	log2(TM10/CM10)	log2(TM10/TM0)	Description
Solyc06g035700.1	Eth	-2.569973739	5.726104117	2.233921858	-6.062155998	ERF025-like
Solyc10g050970.1	Eth	-1.304257551	4.642995359	1.155919346	-4.791333565	ERF109-like
Solyc01g095140.4	Eth	-1.031503824	5.697679565	2.422914962	-4.306268427	ER5 protein
Solyc04g071770.3	Eth	1.046931654	4.148131752	1.415439268	-1.685760831	ERF ABR1-like
Solyc01g108240.3	Eth	-2.985011238	6.732941547	4.101471562	-5.616481223	ERF109
Solyc09g008175.1	IAA	-1.698707053	-1.394928001	-2.072899344	-2.376678397	SAUR71
Solyc11g069093.1	IAA	-1.463058535	-2.139471002	-3.821360577	-3.144948111	auxin-induced protein X15
Solyc05g024260.3	ABA	1.257845286	8.849939626	8.861256981	1.26916264	bidirectional sugar transporter N3
Solyc11g072310.2	GA	2.117350479	3.76016864	2.883632627	1.240814466	gibberellin 20 oxidase 1-like
Solyc05g024260.3	SA	1.257845286	8.849939626	8.861256981	1.26916264	bidirectional sugar transporter N3

**Fig 7:**
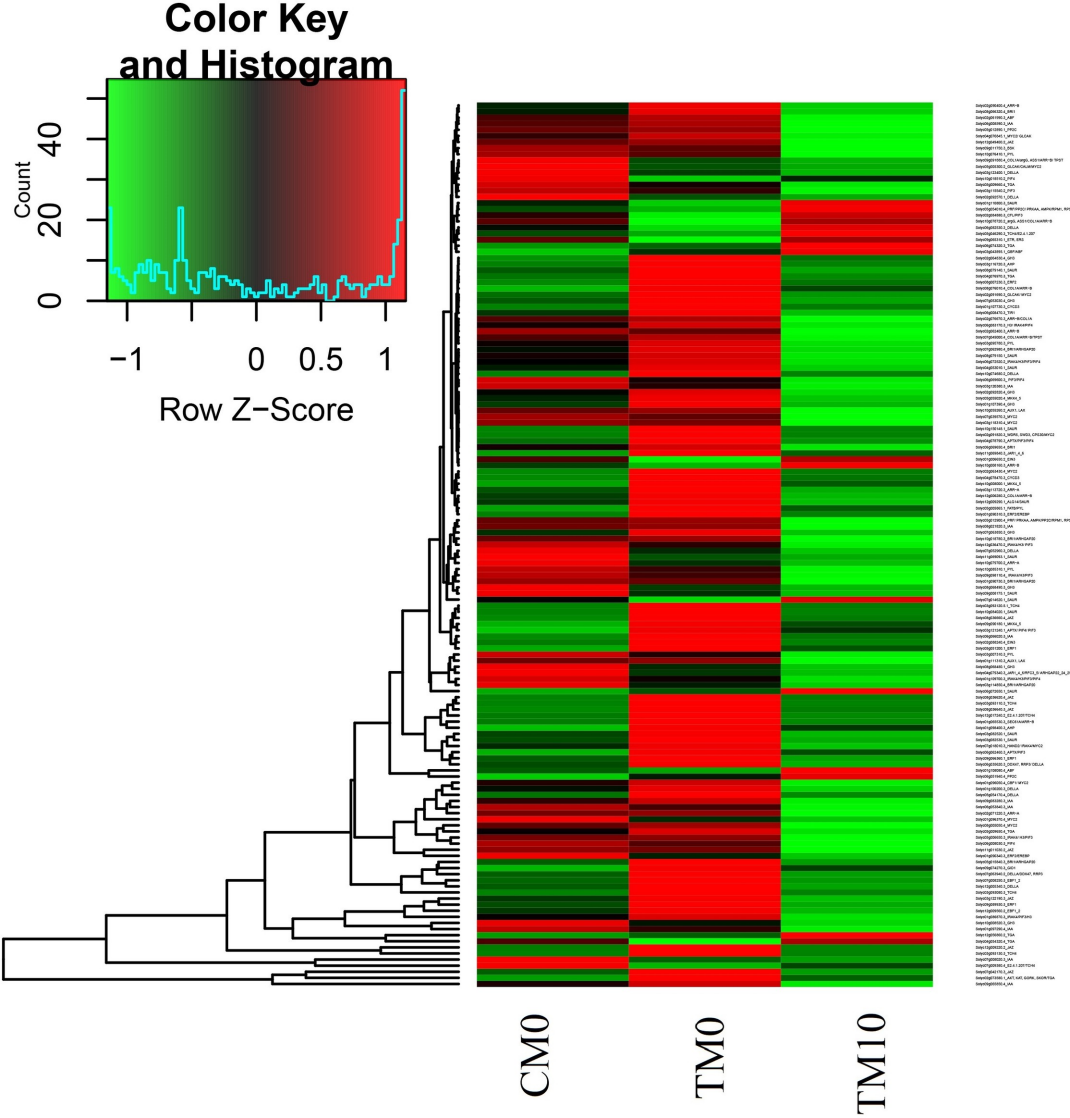
The expression profiles of the DEGs involved in different hormone signaling pathways in groups of CM0, TM0 and TM10.

Notably, similar to DEGs involved in alpha-linolenic acid pathway, most DEGs responded to plant hormone signal transduction were also up-regulated under drought treatment (TM0) when compared with normal condition (CM0). After melatonin irrigation (TM10), the up-regulated expression pattern of these DEGs involved in plant hormone signal transduction was restored to a great extent (Fig 7). On the whole, the expression patterns of DEGs involved in signal transduction of different hormones (IAA, CK, GA, ABA, Eth and JA) have been shown in S4 Fig.

### Classification of DEGs according to transcription factors

In the above mentioned four comparison groups, the DEGs were then classified on the basis of transcription factors (S11–S14 Tables). For example, out of the DEGs from the group of CM10-vs-CM0, nine MYB-related genes, two bHLH-related genes, one NAC-related gene, five MADS-related genes, four WRKY-related genes, 13 AP2-related genes, four C2H2-related genes and one bZIP-related gene were identified (S11 Table). In general, 11 DEGs (one MYB, one bHLH, five AP2, three C2H2 and one bZIP gene) encoded transcription factors common to the four groups (Table 2).

**Table 2:** Transcription factor related DEGs shared by four groups.

Gene ID	TFs	log2(CM10/CM0)	log2(TM0/CM0)	log2(TM10/CM10)	log2(TM10/TM0)	Description
Solyc02g085145.1	MYB	-1.739254824	-2.420415605	-7.389045087	-6.707884305	protein RADIALIS-like 5
Solyc03g114230.2	bHLH	-4.306939333	1.836246718	4.233921858	-1.909264193	bHLH27-like
Solyc06g035700.1	AP2	-2.569973739	5.726104117	2.233921858	-6.062155998	ERF025-like
Solyc10g050970.1	AP2	-1.304257551	4.642995359	1.155919346	-4.791333565	ERF109-like
Solyc03g124110.2	AP2	-2.132834491	3.83902972	2.731385505	-3.240478706	DREB protein 1A
Solyc04g071770.3	AP2	1.046931654	4.148131752	1.415439268	-1.685760831	ABR1-like
Solyc01g108240.3	AP2	-2.985011238	6.732941547	4.101471562	-5.616481223	ERF109
Solyc12g088390.1	C2H2	-1.773507133	-1.046854742	-1.473437274	-2.200089665	zinc finger protein ZAT10-like
Solyc11g073075.1	C2H2	-1.494180824	1.227689588	-1.300679996	-4.022550408	zinc finger protein ZAT11-like
Solyc06g075780.3	C2H2	-1.007553807	2.948485153	2.280398011	-1.675640949	zinc finger protein ZAT1-like
Solyc07g062710.4	bZIP	-1.685218283	-1.945768617	-2.267994922	-2.007444587	basic leucine zipper 61-like

### DEGs analysis under the influence of melatonin

In this study, DEGs impacted by melatonin under normal or drought treatment were focused on for better explaining the role of melatonin in alleviating drought. As mentioned above, without PEG6000 treatment, a total of 447 DEGs were found in tomato leaves treated with 10 μmol/L (CM10) melatonin, when compared with those treated with 0 μmol/L (CM0) melatonin (S2 Table, Fig 3A). Here, protein network diagram was performed to analysis gene/gene function impacted by melatonin under non-drought condition. The result showed that among 447 DEGs, several genes showed closed correlations with other genes. It was worth noting that *Solyc10g079700*.*2* with the most interaction connections had direct interaction with 13 genes, among which 6 belong to MYB gene family and 5 belong to MADS gene family (S5A Fig and S15 Table). On the other hand, with PEG6000 treatment, as mentioned above, the expression of 3258 genes changed significantly under 10 μmol/L melatonin treatment (TM10) when compared to plants only under drought condition (TM0). Among the 3258 DEGs, 350 of them with significantly down-regulation folds, here defined as log2 (TM10/TM0) ≤-4, were selected for gene/gene function analysis with protein network diagram (S3B Fig, S16 Table). The results showed that *Solyc03g116890* and *Solyc05g008020* had the largest number of interacting proteins. In detail, *Solyc03g116890* had direct interaction with six genes of *Solyc03g093610*, *Solyc03g116890*, *Solyc08g036620*, *Solyc08g036640*, *Solyc08g036660* and *Solyc09g083360*. *Solyc05g008020* had direct interaction with six genes of *Solyc02g088670*, *Solyc03g007050*, *Solyc03g116720*, *Solyc04g025005*, *Solyc05g008020* and *Solyc12g005450* (S3B Fig).

**Fig 8:**
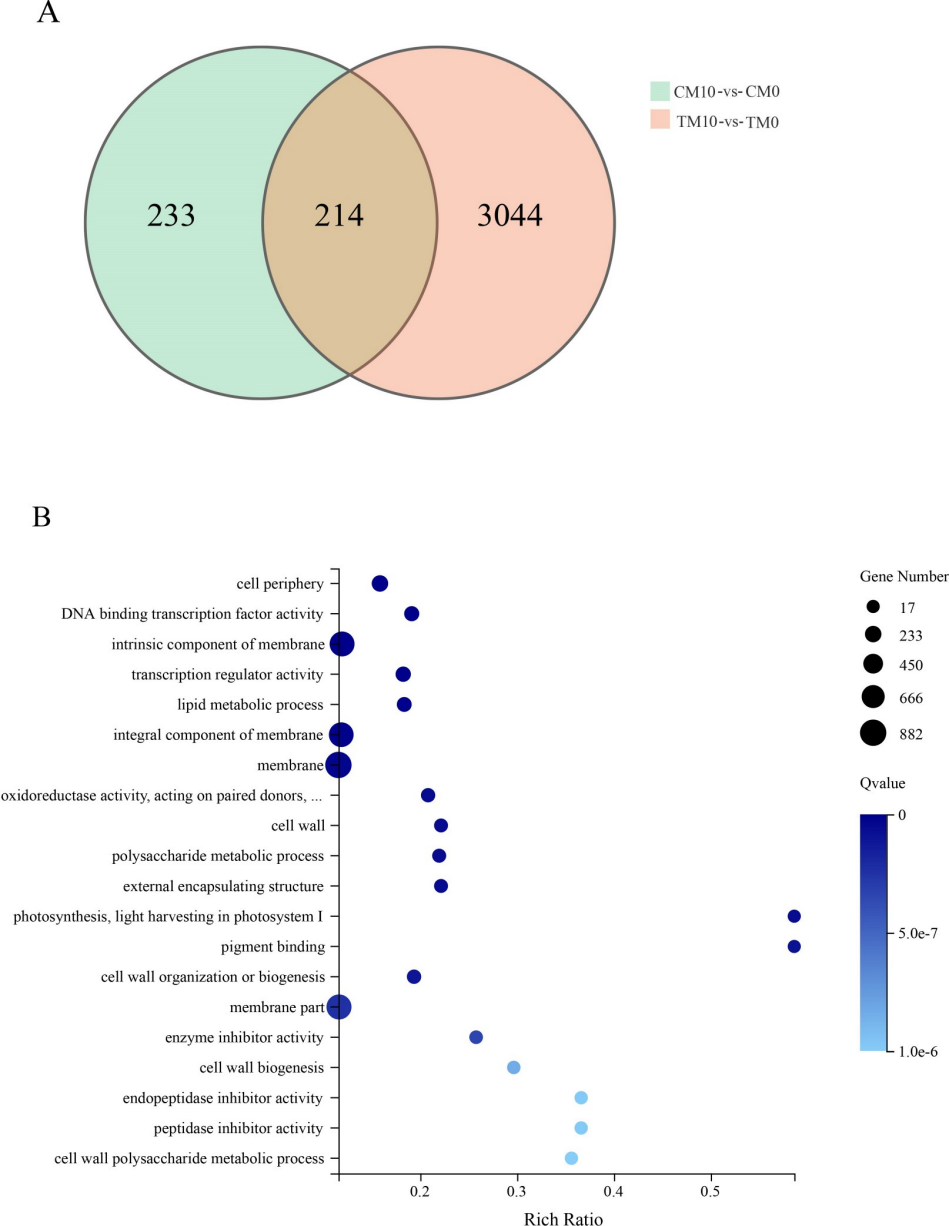
Analysis of DEGs specific to melatonin treatment under drought condition. A. comparative analysis of DEGs between TM10-vs-TM0 and CM10-vs-CM0 comparison groups. The green circle represents DEGs in CM10-vs-CM0, and the red circle represents DEGs in TM10-vs-TM0. B. 3044 DEGs specific belonging to TM10-vs-TM0 were analyzed by GO enrichment and displayed by bubble diagram.

To further compare the effects of melatonin under drought and non-drought conditions from the perspective of DEGs, comparative analysis of DEGs between TM10-vs-TM0 and CM10-vs-CM0 comparison groups were paid attention. The results showed that the two comparison groups had 214 DEGs in common, and number of DEGs specific belonging to TM10-vs-TM0 was 3044 (Figs 3B and 8A). Subsequently, the 3044 DEGs were analyzed by GO enrichment and displayed by bubble diagram (Fig 8B). Among the top 20 GO terms with lowest Qvalue, GO term of “membrane” consisted the maximum number (882) of DEGs (Fig 8B and S17 Table).

### Real-time RT-PCR (RT-qPCR) verification of DEGs

**Fig 9:**
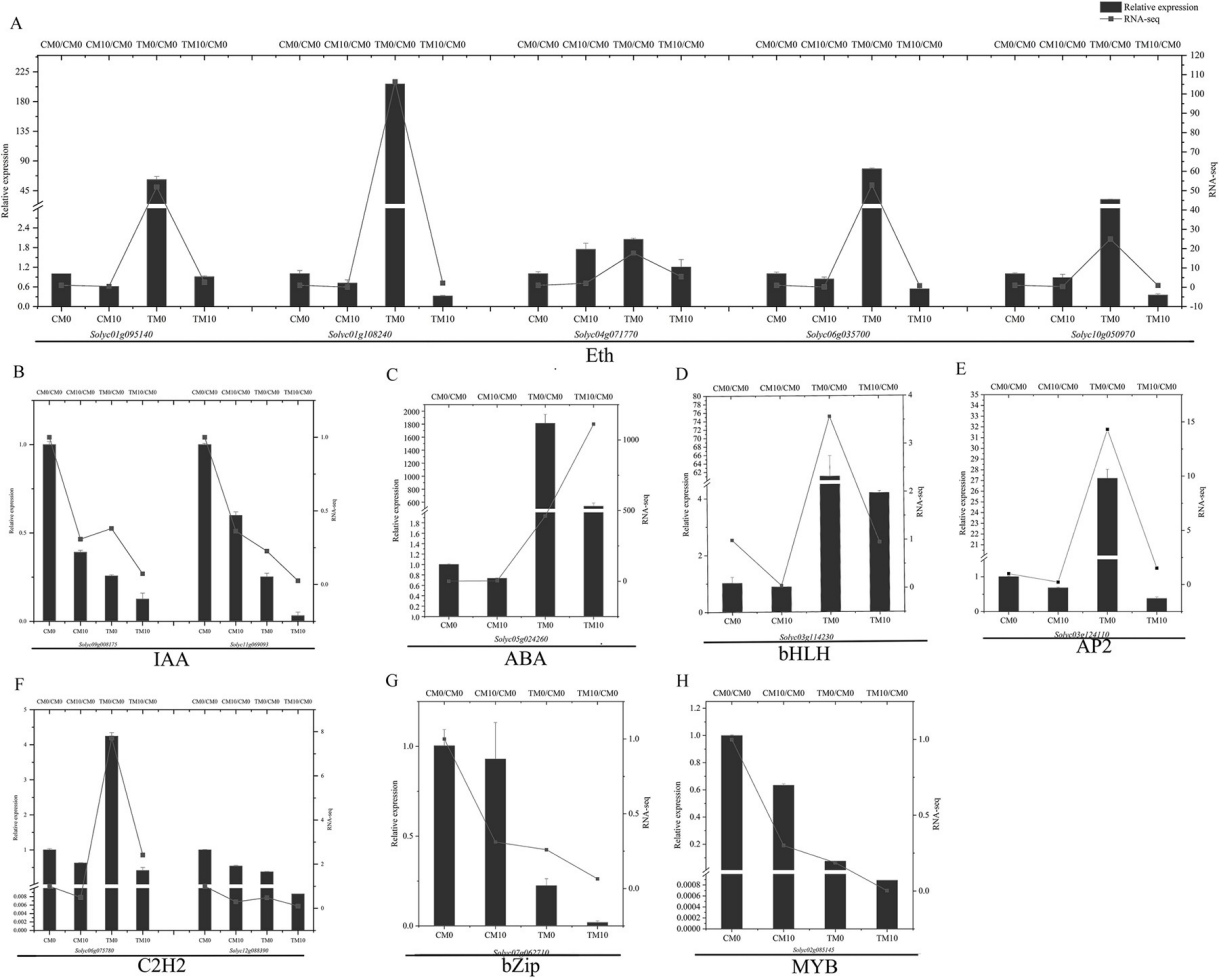
Accompanied by RNA-seq data, verification of phytohormones and transcription factor related DEGs by RT-qPCR with application of different concentrations of melatonin (0, 10 μmol/L) under normal and PEG6000 simulated drought. A. Relative expression and RNA-seq data of five genes related to Eth. B. Relative expression and RNA-seq data of two genes related to IAA. C. Relative expression and RNA-seq data of gene related to ABA. D. Relative expression and RNA-seq data of gene related to bHLH. E. Relative expression and RNA-seq data of gene related to AP2. F. Relative expression and RNA-seq data of two genes related to C2H2. G. Relative expression and RNA-seq data of gene related to bZip. H. Relative expression and RNA-seq data of gene related to MYB. Error bars show the standard error between three biological replicates performed (n = 3).

To validate RNA-Seq data, 14 genes were chosen for real-time RT-PCR (RT-qPCR) analysis (Fig 9). The selected genes comprised eight phytohormone-related and six transcription factors-related genes. The phytohormone-related genes were namely five Eth-related signaling pathway genes *(Solyc01g095140*, *Solyc01g108240*, *Solyc04g071770*, *Solyc06g035700* and *Solyc10g050970*), two IAA-related signaling pathway genes (*Solyc09g008175* and *Solyc11g069093*), and one ABA-related signaling pathway gene (*Solyc05g024260*). The transcription factor-related genes consisted of one bHLH- (*Solyc03g114230*), one AP2- (*Solyc03g124110*), two C2H2- (*Solyc06g075780* and *Solyc12g088390*), one bZip- (*Solyc07g062710*) and one MYB-related gene (*Solyc02g085145*). The RT-qPCR results were broadly consistent with the RNA-Seq data for the majority of the tested genes. For example, relative expression of *Solyc01g095140* in CM0, CM10, TM0 and TM10 was 1.00, 0.61, 61.78 and 0.91, respectively. Then, converting the data of log2(CM10/CM0), log2(TM0/CM0), log2(TM10/CM10) and log2(TM10/TM0) derived from RNA-seq data (Tables 1 and 2) to CM0/CM0 (1.00), CM10/CM0 (0.49), TM0/CM0 (51.90) and TM10/CM0 (2.62) for mapping. In both RNA-Seq and RT-qPCR data, *Solyc01g095140* had the highest and lowest expression in TM0 (+PEG6000/−melatonin) and CM10 (−PEG6000/+melatonin) plants, respectively.

## Discussion

Tomato (*Solanum lycopersicum* L.) is widely cultivated all over the world as one of the most important horticultural crops. However, tomato plants are highly susceptible to water deficit, not only during the vegetative growth phase but particularly during the stages of flowering and the fruit set. Water deficiency seriously damages leaf and fruit size, fruit number, photosynthetic rate and fruit quality [40, 41]. Melatonin is a pleiotropic signaling molecule; in addition to promoting ripening and improving the quality of tomato fruit postharvest, it also provides physiological protection for plants against various abiotic stresses [19]. Researchers found that exogenous melatonin application improves tolerance to water deficit by promoting cuticle formation in tomato plants [33]. In the current study, the mechanism by which melatonin alleviated drought damage in tomato plants was investigated from the perspective of photosynthetic protection and differentially gene expression induced by melatonin application.

Tomato plant height decreased significantly after simulation of drought stress by continuous PEG6000 treatment, which indicated that drought delayed the growth rate of tomato plants (Fig 1). These results were also consistent with those from a previous study with a different tomato cultivar, Jing fen 2, which found that, relative to plants grown with drought stress, plant height under mild stress, moderate stress or severe stress decreased by 11.49%, 28.6% or 43.98%, respectively [42]. Another recent study showed that water deficit was associated with significant inhibition of growth of tobacco seedlings, decreasing the biomass of shoots and roots, and that melatonin application could alleviate drought stress-induced growth inhibition [43]. In the present study, relative to plants grown without melatonin application, tomato plant height increase ratio was enhanced from day 5 to day 7 after irrigation with melatonin (Fig 1B).

Plants growing under drought conditions face many challenges at both the biochemical and molecular levels, which eventually hinder the growth and yield of plants [44, 45]. The plant photosynthetic apparatus will be destroyed under drought stress, resulting in decreases in photosynthetic rate, stomatal conductance, transpiration rate, PSII photochemical efficiency and photosynthetic electron transfer rate. However, melatonin prevents the photosynthetic mechanism from being damaged by drought, and then restores the photosynthetic efficiency of plants [46]. In the current study, decreased levels of Fv/Fm, Fv’/Fm’, ETR and qP caused by drought stress were largely recovered following melatonin treatment (Fig 2). These results were consistent with those from the previous study where the recovered photosynthetic rate as a result of melatonin treatment of droughted plants was accompanied by improved Fv/Fm and ETR values [46].

Previous similar research reports showed that co-occurrence of cold and drought stress can alter the response of tomato plants at morphological, physiological and molecular levels, which finally affect crop production [47]. In order to reveal the comparison results of DEGs among multiple groups of treatments under individual stress (drought), pairwise comparisons were performed in four groups of CM10-vs-CM0, TM0-vs- CM0, TM10-vs-CM10 and TM10-vs-TM0. Numbers of DEGs and venn diagram analysis are illustrated in Fig 3. The comparative group of TM10-vs-CM10 contained the largest number of DEGs. Also, compared with the group of TM0-vs-CM0 which contained much more up-regulated DEGs, the number of up-regulated DEGs was only slightly higher than that of down-regulated DEGs in the group of TM10-vs-CM10 (Fig 3). These results hinted that melatonin treatment increased both the number of total DEGs and down-regulated DEGs. Similarly, Debnath et al. (2020) showed that exogenous melatonin application protected tomato seedlings from acid rain stress by changing the expression patterns of DEGs, principally secondary metabolites and transcription factor-related genes [48].

Function annotation of DEGs were often performed with GO or KEGG analysis [38, 49]. In this study, DEGs from the four groups were classified with GO and KEGG database (Figs 4 and 5). The GO analysis in drought treatment (TM0-vs-CM0) showed that these genes were significantly enriched in the following GO terms: metabolic process, cellular process, membrane, organelle, binding, catalytic activity. These GO terms were as same as GO analysis of melatonin treatment (CM10-vs-CM0). These results suggested that the genes related to membrane, regulation and catalytic activity were both effected by drought and melatonin treatment. In other word, melatonin may increase the tolerance of drought by reduced the damage of membrane which caused by drought. KEGG pathway analysis were also performed to elucidate the significance of DEGs. The DEGs between melatonin and non-melatonin under drought treatment revealed more information about the effect of melatonin. In TM10-vs-TM0 group, the DEGs were mainly enriched in “plant hormone signal transduction”, “photosynthesis-antenna proteins”, and “linoleic acid metabolism” (Fig 5).

Linolenic acid can reduce structural and functional damages of cellular membranes caused by physiological stresses via increasing the fluidity of the cell membrane and modulating related signal transduction pathways [50–52]. In our study, transcripts of eight genes related to linolenic acid were significantly regulated in leaves treated with melatonin (Fig 6). In detail, when compared with TM0, DEGs related to linoleic acid synthesis (TGL4) and catabolism related enzymes (CYP1A2 and LOX2S) in TM10 were -up and -down regulated, respectively. These results were consistent with other report in which linolenic acid metabolism-related genes were also significantly regulated in the leaves of maize seedlings treated with exogenous melatonin [53]. So, it can be inferred that drought stress could be relieved by melatonin which may help releasing linolenic acid from the membrane lipids of leaves through down-regulation of genes related to LOX and CYP1A2.

Phytohormones and transcription factors play critical roles in the response of plants to drought stress. Crosstalk between melatonin and other phytohormones such as IAA, GA, CTK, JA, ETH and ABA have been demonstrated and a large number of genes participated in various hormone signaling pathways could be regulated by melatonin [54]. In the current study, DEGs involved in different hormone signaling pathways were identified from the four groups (S7–S10 Tables). For instance, ethylene is a hormone closely related to the processes of maturity, senescence and stress response. Ethylene-responsive factors (ERFs, belonging to the AP2/ERF family) are widely involved in the regulation of gene expression in response to external environmental stresses in plants. The expression of ERFs increased under drought stress to achieve increased stress tolerance [55]. In tomato, ER5 and ERF5 have been shown to function in promoting adaptation to drought stress in tomato [56, 57]. In the current study, five ethylene-related DEGs, which were common to the four comparative groups (CM10-vs-CM0, TM0-vs-CM0, TM10-vs-CM10 and TM10-vs-TM0), contained *ER5* (*Solyc01g095140*) and four *ERF* genes (*Solyc06g035700*, *Solyc10g050970*, *Solyc04g071770* and *Solyc01g108240*) (Tables 1 and 2). The expression of these genes was up-regulated by drought, whereas, interestingly, melatonin irrigation reduced the up regulatory effect of these genes in response to drought (Table 1, Fig 9). Similar results were observed during melon root development; compared with the group of melon roots exposed to copper stress, the expression of the *AP2/ERF* gene in the melatonin pre-treatment group was down regulated and the authors speculated that melatonin may alleviate copper stress and promote melon root development by reducing the expression of the *AP2/ERF* gene [58]. In fact, our results showed that most of DEGs related to hormones were up-regulated after drought treatment and returned to normal level after melatonin treatment (Fig 7).

Auxin regulates the expression of hundreds of genes, such as members of the early auxin response gene family, Aux/IAA, Gretchen Hagen3 (GH3) and Small Auxin-Up RNA (SAUR) [59]. In the present study, two IAA-related genes, *Solyc09g008175* (*SAUR71*) and *Solyc11g069093* (*auxin-induced protein X15*), which were common to the four groups, showed similar expression patterns (Table 1, Fig 9). The expression of these two genes was suppressed under PEG6000 treatment, whereas melatonin treatment further enhanced the down-regulation effect on gene expression (Fig 9). Similar results were reported in a previous study which revealed that the *SAUR* gene family contained 99 members and that the expression of many *SAUR* genes, including *SAUR71*, was down-regulated under drought treatments [60]. High levels of endogenous SA and the application of exogenous SA can promote stomatal closure and induce reduced photosynthesis rate during water deficit to conserve water [61]. The *Solyc05g024260* gene is related to both SA and ABA signal transduction and is shared by the four groups (Table 1). The expression of *Solyc05g024260* was up-regulated by drought, whereas melatonin irrigation reduced the drought-induced up-regulatory effect (Fig 9).

In the current study, DEGs associated with different types of transcription factors (TFs) were identified from the four groups (S11–S14 Tables). A previous study had reported that expression of a gene named *IbZFP1*, encoding a C2H2 TF, could be induced by PEG treatment in sweet potato [62]. C2H2 zinc-finger proteins regulate plant responses to drought stress through ABA-dependent and ABA-independent pathways [63]. In the present study, three different C2H2 gene family members (*Solyc12g088390*, *Solyc11g073075* and *Solyc06g075780*) were shared by the four DEG groups (Table 1). The expression of *Solyc06g075780*, but not that of *Solyc12g088390*, was induced by PEG6000 treatment, but the expression of both genes could be down-regulated by melatonin treatment (Fig 9). Mitogen-activated protein kinase (MAPK) cascade pathways play a crucial role in regulating the biological processes of plants exposed to abiotic stresses, such as drought [64]. The expression pattern of MAPKs showed up-regulation by melatonin under drought stress, accompanied by the up-regulation of expression of several families of TFs, such as WRKY, DREB and MYB, which are the main components of the MAPK signaling pathway in plants under stress conditions [64, 65]. Recent research has shown that melatonin changes the expression of many transcription factor genes and secondary metabolite genes in tomato to improve the plant’s tolerance to acid rain stress [48]. In the present study, different types of transcription factors were found among the DEGs (Table 2, Fig 9).

In brief, melatonin was involved in improving plant stress tolerance and growth under drought stress conditions. In the present study, in combination with the analysis of several physiological parameters (Fv/Fm, Fv’/Fm’, ETR, qP, qN and NPQ), RNA-Seq technology was used to investigate the effect of response to melatonin irrigation under non-drought and drought conditions. The analysis of plant phenotype, physiological parameters and DEGs among different comparison groups provides a firm foundation to the physiological and molecular mechanisms of the role of melatonin in improving tomato drought stress tolerance.

## Supporting information

S1 Fig Flow chart of RNA-Seq analysis. (TIF)

S2 Fig The volcano plot of DEGs in the four comparison groups (CM10-vs-CM0, TM0-vs-CM0, TM10-vs-CM10, TM10-vs-TM0). (TIF)

S3 Fig Function annotation of DEGs in the four comparison groups (CM10-vs-CM0, TM0-vs-CM0, TM10-vs-CM10, TM10-vs-TM0) by KEGG analysis. (TIF)

S4 Fig Response in plant hormone signal transduction to exogenous melatonin treatment under drought stress. The proteins in marked in green indicates down-regulated genes, the proteins marked in blue indicates genes that are both up-regulated and down-regulated.
(TIF)

S5 Fig Protein network diagram of DEGs in CM10-vs-CM0 and TM10-vs-TM0. (TIF)

S1 Table Primer sequences used for quantitative real-time PCR. (PDF)

S2 Table Total CM10 vs CM0 DEGs. (CSV)

S3 Table Total TM10 vs TM0 DEGs. (CSV)

S4 Table Total TM0 vs CM0 DEGs. (CSV)

S5 Table Total TM10 vs CM10 DEGs. (CSV)

S6 Table DEGs with the greatest expression changes in TM10 vs TM0. (XLS)

S7 Table Plant hormone related DEGs in the comparison group of CM10 vs CM0. (XLSX)

S8 Table Plant hormone related DEGs in the comparison group of TM0 vs CM0. (XLSX)

S9 Table Plant hormone related DEGs in the comparison group of TM10 vs CM10. (XLSX)

S10 Table Plant hormone related DEGs in the comparison group of TM10 vs TM0. (XLSX)

S11 Table Transcription factor related DEGs in the comparison group of CM10 vs CM0. (XLSX)

S12 Table Transcription factor related DEGs in the comparison group of TM10 vs CM0. (XLSX)

S13 Table Transcription factor related DEGs in the comparison group of TM10 vs CM10. (XLSX)

S14 Table Transcription factor related DEGs in the comparison group of TM10 vs TM0. (XLSX)

S15 Table Information of 13 genes which has direct interaction with *Solyc10g079700*. (CSV)

S16 Table DEGs of log2 (TM10TM0) ≤-4 in TM10 vs TM0. (CSV)

S17 Table 882 DEGs in membrane term of TM10 vs TM0. (CSV)
