# Supplementary figures and images for: Transcriptome and physiological analysis of increase in drought stress tolerance by melatonin in tomato
Source: PLoS One. 2022 May 17;17(5):e0267594. doi: 10.1371/journal.pone.0267594 (PMC9113596; doi:10.1371/journal.pone.0267594)

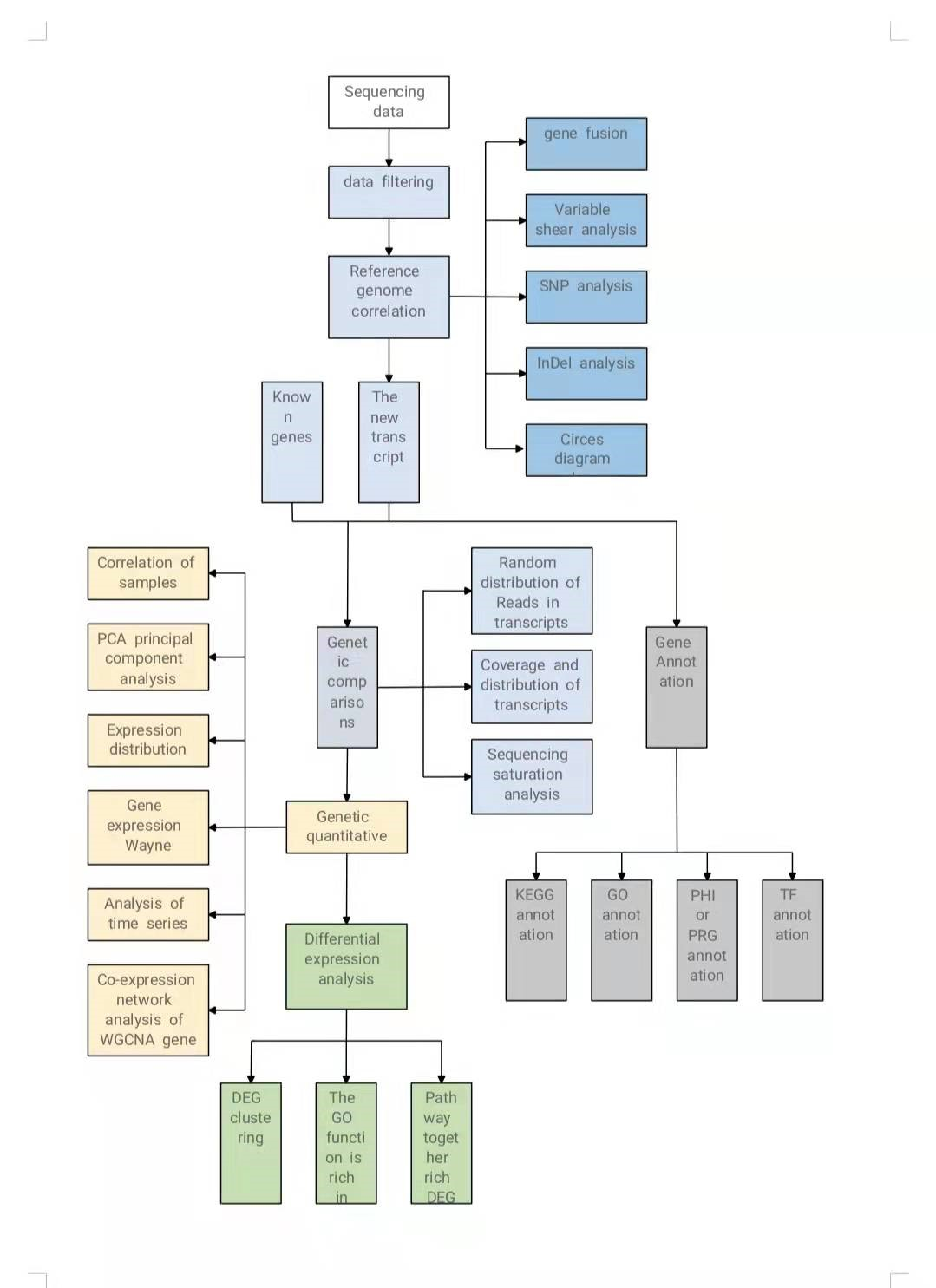

Supplement: S1 Fig — (TIF) [file pone.0267594.s001.tif]

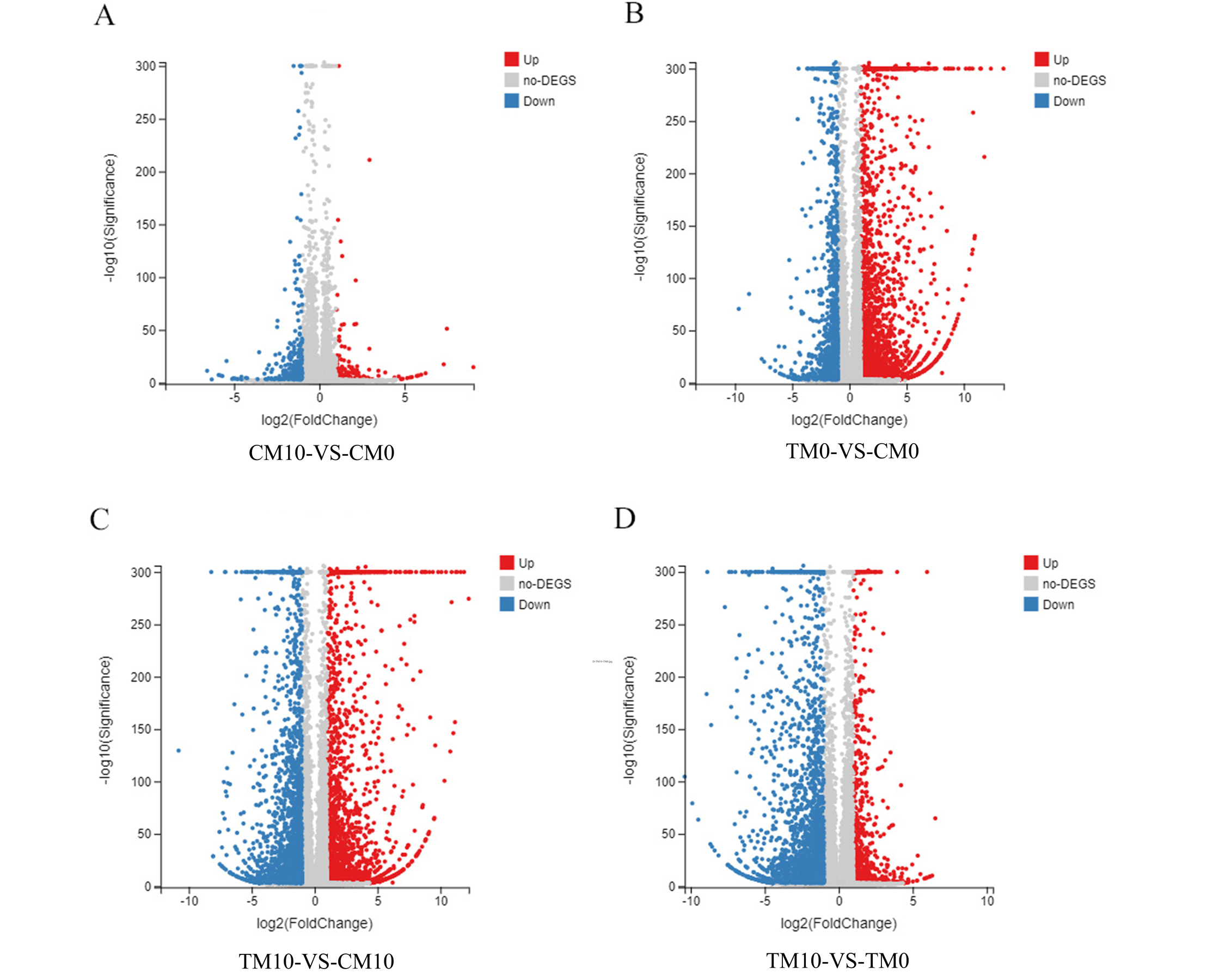

Supplement: S2 Fig — (TIF) [file pone.0267594.s002.tif]

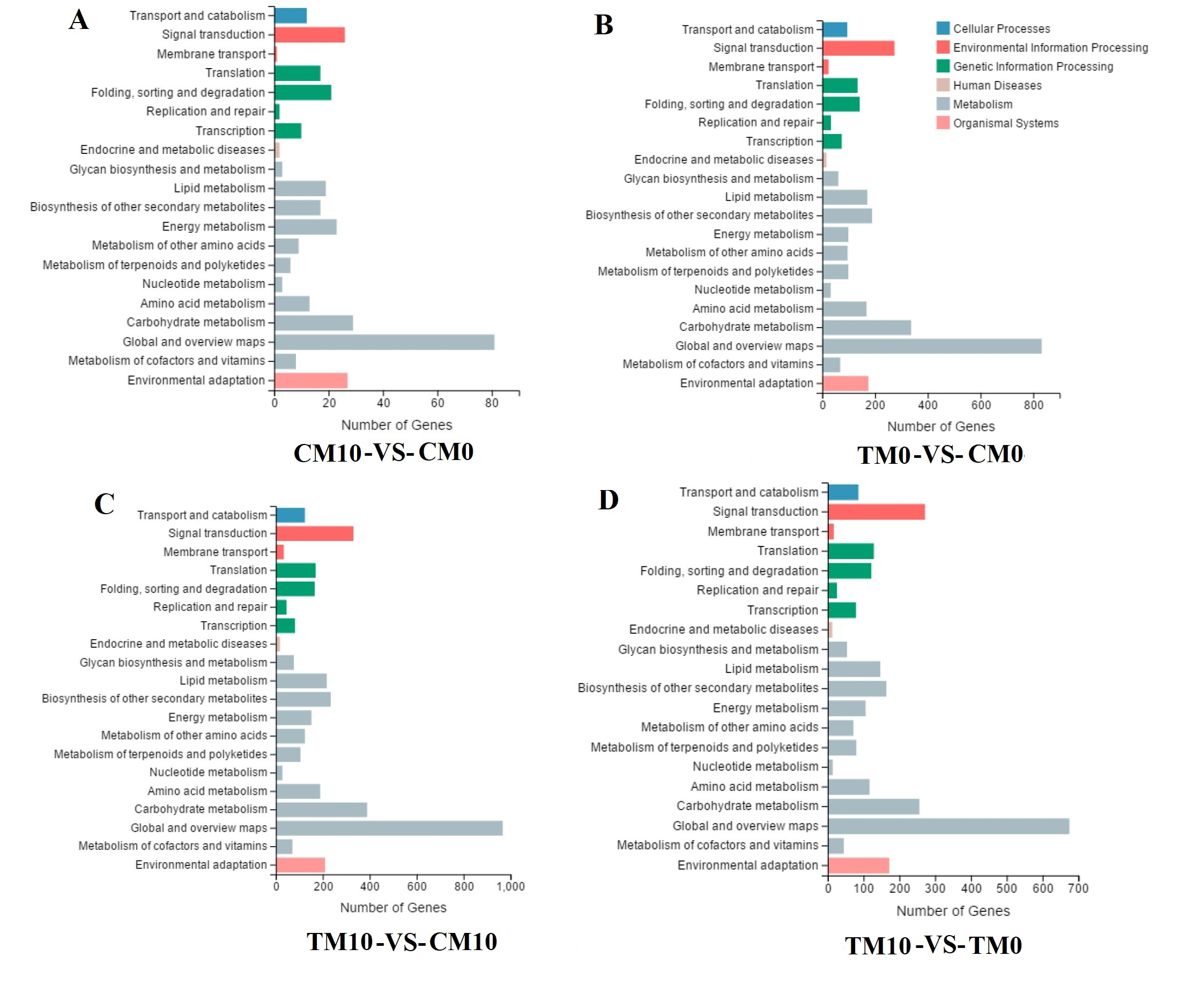

Supplement: S3 Fig — (TIF) [file pone.0267594.s003.tif]

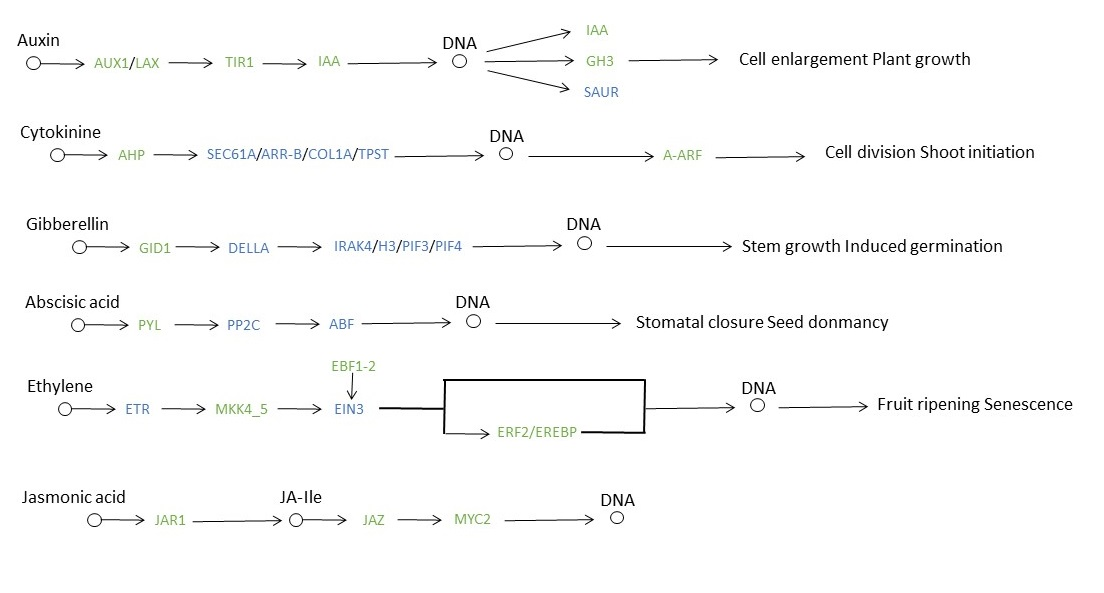

Supplement: S4 Fig — The proteins in marked in green indicates down-regulated genes, the proteins marked in blue indicates genes that are both up-regulated and down-regulated. (TIF) [file pone.0267594.s004.tif]

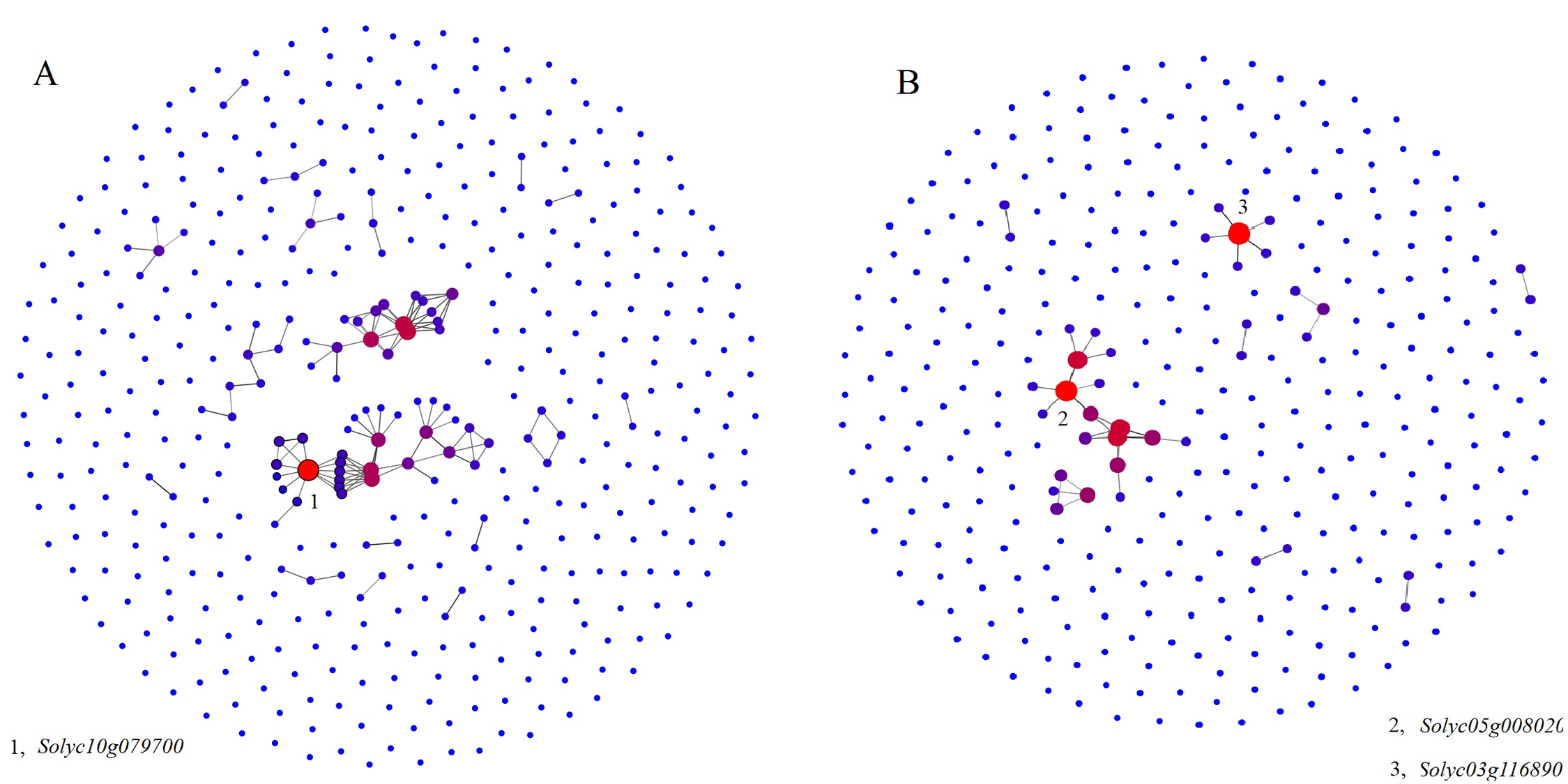

Supplement: S5 Fig — (TIF) [file pone.0267594.s005.tif]
