# Supplementary material for: Transcriptome and physiological analysis of increase in drought stress tolerance by melatonin in tomato
Source: PLoS One. 2022 May 17;17(5):e0267594. doi: 10.1371/journal.pone.0267594 (PMC9113596; doi:10.1371/journal.pone.0267594)
Supplement: S1 Table — (PDF) [file pone.0267594.s006.pdf]

Table S1 Primer sequences used for quantitative real-time PCR

Name	Forward primer Sequence (5' →3')	Reverse primer Sequence (5' →3')	Description
SlActin	TGTCCCTATCTACGAGGGTTATGC	AGTTAAATCACGACCAGCAAGAT	Reference gene
Solyc01g095140	AGCGATGGATTTCGTGTCTG	TACTATAACCCTGCCGGAGC	Eth
Solyc01g108240	TTTACCGTATGCAGAGTCCA	CAACCCGAAAATGAATAGAC	Eth
Solyc04g071770	CCGTATGAACAGTCGCAATC	TATCCGGCCTGTAACCCCAC	Eth
Solyc06g035700	CATACTCCTACCCTGTCCCTG	CCTCTGCCATATCATCAAGC	Eth
Solyc10g050970	AGATTGTTGGGAAGACGAAA	TAGCTCTTGGACCTCTAAAT	Eth
Solyc09g008175	GCCAAATGATGTGAAAGAAG	AATTCCGCAGCACGACAAGG	IAA
Solyc11g069093	CCGAGGACGTTAAAGAGGGT	TCACTTGGCCTGCATGGTAC	IAA
Solyc05g024260	TGCTATCATGTGGTTTGGCTAT	AATCTGCTCCGAGTTTACTG	ABA
Solyc03g114230	GCCTTCAACATCTTGTAGCA	GTAAACCTTATCGCCGATTG	bHLH
Solyc03g124110	CAAATATGGCAGAAGGACTC	TTGTTACTACGCATTTACCT	AP2
Solyc06g075780	TGGGTCAGGCTTTAGGTGGA	TGGAGGTACAGGTGGCATAG	C2H2
Solyc12g088390	ATGGACCAAAGGCAAGATTA	ATCCAAAACACTTACCACAAAC	C2H2
Solyc07g062710	ATAGCGCACTCAGGCAACGA	CGCTTCTTCCGTACAACTCA	bZip
Solyc02g085145	TCAAATTCACTCACTGCTTGG	CACCAGATTCAATGTGCTTA	MYB
